# Clinical and immunological failure among HIV-positive adults taking first-line antiretroviral therapy in Dire Dawa, eastern Ethiopia

**DOI:** 10.1186/s12889-019-7078-5

**Published:** 2019-06-17

**Authors:** Getinet Abera Lenjiso, Berhanu Seyoum Endale, Yadeta Dessie Bacha

**Affiliations:** 1 Disease Prevention and Control Case Team, Dire Dawa Administration Health Bureau, P.O Box 1377, Dire Dawa, Ethiopia; 2 Department of Medical Laboratory Science, Haramaya University Colleges of Health and Medical Sciences, Harar, Ethiopia; 3 School of Public Health, Haramaya University Colleges of Health and Medical Sciences, Harar, Ethiopia; 4 Armauer Hansen Research Institute, Addis Ababa, Ethiopia

**Keywords:** Clinical failure, Immunological failure, Adherence, Functional status

## Abstract

**Background:**

Access to antiretroviral therapy (ART) in Ethiopia has been scaled up since the introduction of the service in 2003. Free ART was launched in 2005, resulting in fewer new human immunodeficiency virus (HIV) infections and deaths from acquired immunodeficiency syndrome (AIDS). However, immunological and clinical failures for first-line ART due to poor adherence and other factors have received less attention. Thus, this study aims to determine the magnitude and associated factors of clinical and immunological failure among HIV-positive adults after six months of first-line ART in Dire Dawa, Eastern Ethiopia.

**Methods:**

A facility-based cross-sectional study was conducted using secondary data of patients on ART in all health facilities providing ART services in Dire Dawa. A total of 949 samples were collected. The data were entered into Epidata version 3.02, and the analysis was performed using SPSS version 16.0. Univariate and multivariate analyses were performed to determine the magnitude of clinical and immunological failure and identify factors significantly associated with the outcome variable.

**Results:**

The magnitude of clinical and immunological failure was 22.7% (*n* = 215). Of these, 33 (15%) patients were switched to second-line ART. CD4 count ≤100 cells/mm3 (AOR: 1.78, 95% CI: 1.18–2.69), poor adherence (AOR: 2.5, 95% CI: 1.19–5.25), restarting after interruption of ART (AOR: 1.93, 95% CI: 1.23–3.07), regimen change (AOR: 1.50, 95% CI: 1.05–2.15), ambulatory/bedridden functional status at the last visit on ART (AOR: 2.41, 95% CI: 1.22–4.75) and patients who died (AOR: 3.94, 95% CI: 1.64–9.45) had higher odds of failure.

**Conclusion:**

The magnitude of clinical and immunological failure was high. To curb this problem, initiation of ART before the occurrence of severe immune suppression, early detection and management of failure and improved adherence support mechanisms are recommended. Restarting treatment after interruption and regimen changes-should-be-made-cautiously.

## Background

At the beginning of the AIDS pandemic, treatment was confined to palliative care and management of opportunistic infections (OIs). The advent of ART has made HIV a manageable chronic illness; however, there is still no cure [1]. From 1995 to 2010, ART has saved 14 million life years in low- and middle-income countries, including 9 million in sub-Saharan Africa (SSA) [2].

In Ethiopia, national guidelines on the use of antiretroviral drugs (ARVs) were developed, and the ART program was launched in 2003. Subsequently, in 2005, a free ART program was initiated in three government hospitals in Addis Ababa. Since then, the geographic distribution and number of centers with ART services have increased to 743 health facilities in 2010/11 [3]. A 53% decline in AIDS-related death has been reported between 2005 and 2011 [2].

The primary goals of HIV treatment include suppression of viral replication, restoration of the immune response, a halt in the progression of disease, increased survival rates, reduced morbidity, and a better quality of life [4]. For ART, a high level of sustained adherence is necessary to suppress viral replication and improve immunological and clinical outcomes, decrease the risk of developing ARV drug resistance, and reduce the risk of transmitting HIV [5]. Failure to reduce the viral load can lead to the evolution of drug resistance, with subsequent immunological and clinical failures [6].

Although estimates suggest that only 2% of those currently on ART are on a second-line regimen worldwide, a far greater number are likely to be failing virologically but have not switched from first-line therapy [7]. The world health organization (WHO) estimated that close to 500,000 to 800,000 patients required switching to second-line regimens by 2010 [8].

Identifying patients failing on first-line treatment is a major challenge in ART programs in resource-limited settings. As a result, patients who are failing on a first-line regimen are often not switched to a second-line regimen in a timely manner [9]. This leads to high morbidity and mortality among patients who fail on first-line ART [9, 10].

The time of switching is dictated by treatment failure, and this can be measured in three ways: clinically, immunologically, and virologically. Viral load monitoring is the gold standard method to diagnose ART failure [9]. The sensitivity and specificity of the WHO-based clinical and immunological failure algorithm were 31 and 87%, respectively, which would result in 14% of those with viral suppression being switched inappropriately to second-line ART [11]. However, due to the lack of access to viral load determination in Ethiopia, the diagnosis of treatment failure has been mainly performed by clinical and immunological assessment methods before 2017. To substantially increase the success rate of ART, there is a need to know the magnitude of clinical and immunological failure for first-line ART and to better understand the factors associated with it.

Monitoring for early warning signs of failure should be intensified to prevent resistance to ARVs, one of which is through the identification of clinical and/or immunological failure, which are surrogate markers to the presence of virological failure [12]. Different studies describing the prevalence and predictors of immunological and virological failures have been conducted in different countries. However, there is scarcity of data in Ethiopia in general and in our study area in particular. A single study conducted in Ethiopia showed a 21% prevalence of immunological failure [13]. Therefore, the present study was conducted to determine the magnitude of clinical and immunological failure and associated factors among HIV-positive adults after six months of first-line ART in Dire Dawa, Eastern Ethiopia.

## Methods

### Study area

The study was conducted in Dire Dawa administration, which is located in the eastern part of Ethiopia 515 km away from Addis Ababa. Thirteen health facilities, including 9 health centers and 4 hospitals, provide ART services. As per the regional health bureau report at the end of August 2013, a total of 7569 clients had been started on ART. The proportion of patients who had ever been started on ART at a public hospital, 9 health centers, and 3 private hospitals were 61, 29 and 10%, respectively. The procedure of enrollment in chronic HIV care in the administration was that patients who tested positive for HIV at different service outlets were enrolled in HIV care and registered as pre-ART. The medical eligibility criteria for ART initiation and preferred first-line regimens were as per the national guideline [14].

### Study design

Health facility-based cross-sectional study design using secondary data was used. The study was conducted in all ART providing health facilities in Dire Dawa, Eastern Ethiopia, from January 1 to 30, 2014. Out of the total 7569 people living with HIV (PLHIV) who were put on treatment at the time of the study, 7306 of them were adults and adolescents above the age of 14 years.

The study population was HIV-positive adults and adolescents (age ≥ 15 years) who started on ART in the study area and took ART for more than 6 months. The records of all HIV-positive adults and adolescents who were taking ART for more than 6 months from the facility they started the treatment between December 2003 and January 2013 were included in the study.

### Measurements

The dependent variable was clinical and/or immunological failure among HIV-positive adults and adolescents on the first-line ART 6 months after ART initiation. Clinical failure is the occurrence of new or recurrent WHO stage IV condition 6 months after ART initiation. Immunological failure occurs when there is a fall of CD4 counts to pretherapy baseline (or below) or 50% fall from the on-treatment peak value (if known) or persistent CD4 levels below 100 cells/mm^3^ 6 months after ART initiation [14, 15]. The independent variables were sociodemographic characteristics, disclosure, duration on ART, eligibility criteria for ART initiation, CD4 level, WHO clinical staging, functional status, history of tuberculosis (TB), type of ARV regimens, history of treatment interruption, prior exposure to ARVs, substitution of first-line ART, switch to second-line ART, adherence, outcome of care, etc.

### Sample size and sampling techniques

The sample size was calculated using a single population proportion formula considering 95% significance level, 21% magnitude of treatment failure [13], 2.5% degree of precision, and 10% nonresponse rate considering for compensation of incomplete records. The final sample size calculated was 989. Based on this sample size, all health facilities providing ART service were included, and the sample size was proportionally allocated to each facility based on the patient load the health facilities had. The sampling frame was that the list of all patient records in the specified period was obtained from the patient registers and electronic databases in all the health facilities. The lists of patients were then stratified based on the duration after ART initiation in each health facility, and the final sampling frame was prepared. Stratified proportionate sampling with a systematic random sampling method was then used to obtain the samples.

### Data collection

Data collection was carried out using a pretested structured data extraction tool. Pretesting of the data extraction tool was conducted on 5% of the sample size in a hospital outside the study area. The data extraction tool was developed from the data elements on the nationally standardized HIV patient intake and follow-up formats, which are prepared based on the WHO patient monitoring guidelines [16]. The data were collected by 12 data collectors who had a minimum diploma in information and communication technology (ICT). After obtaining permission from the medical directors, all the data were collected from the individual patient chart after the data extraction tool was provided to the data collectors and charts brought to them from the medical record units. Three supervisors were involved with the responsibility to collect the data extraction tools every day after checking the completeness and consistency. The data collectors were trained in ART data management and familiar with all the information in the data extraction instrument. Orientation was also given to the data collectors and supervisors. The collected data were reviewed after the initial data entry and after the final SPSS data file was created.

### Statistical analysis

The collected data were entered into Epi-data version 3.02 and exported to SPSS version 16.0 for cleaning and analysis. Univariate analysis was used to determine the magnitude of treatment failure and describe the data. Bivariate analysis was performed to estimate the magnitude of the association with treatment failure. A binary logistic regression model was used to identify covariates that were associated with the outcome variable (treatment failure). Factors that were marginally associated at the *p*-value **<** 0.10 in the bivariate analysis were included in the multivariable model. A significance level of 0.05 was used to guide the interpretation of relationships in the final multivariable model calculating AOR and 95% CIs. Variables with more than 10% missing values due to incomplete recording on patient charts were excluded from the analysis.

Ethical clearance was obtained from the Institutional Research Ethics Review Committee of Haramaya University, College of Health and Medical Sciences. Support letter was obtained from the Dire Dawa administration health bureau. Informed consent from the health facilities medical directors was also obtained. The information was kept confidential and anonymous.

## Results

### Sociodemographic characteristics

A total of 949 study participants were included. The majority of the respondents (63%) were females. The median age of the patients at the start of ART was 32 years (IQR: 28–40). Nearly all (95%) of the patients resided in Dire Dawa town. Four hundred two of the patients (43%) were married or cohabitating. The majority of the patients (65%) were Orthodox Christians by religion. Four hundred twelve (44%) of the patients had a primary level of education. Nearly two out of three (65%) patients were unemployed at the time of enrollment in chronic HIV care. Six hundred fifty-eight (69%) of the patients disclosed their HIV status to someone they had a close relationship with. The distribution of survey patients across the health facility type showed that the majority of the study subjects (60%) were from the public hospital, 288 (30%) were from health centers, and 98 (10%) were from private hospitals (Table 1).

**Table 1:** Sociodemographic characteristics of the study subjects in Dire Dawa, Eastern Ethiopia, January 2014

Sociodemographic characteristics	Frequency	Percent (%)
Sex		
Male	352	37
Female	597	63
Age (in years)		
15–24	108	11
25–34	402	42
35–44	281	30
45–54	120	13
55^+^	38	4
Median age 32 (IQR: 28–40)		
Place of residence		
Dire Dawa	902	95
Outside of Dire Dawa	47	5
Marital Status (n = 937)		
Never married	157	17
Married or cohabitating	402	43
Divorced or separated	239	26
Widowed/er	139	15
Religion (n = 939)		
Orthodox	614	65
Muslim	244	26
Others^*^	81	9
Educational status (*n* = 934)		
No formal education	210	22
Primary	412	44
Secondary	258	28
Tertiary	54	6
Employment (*n* = 855)		
Employed	266	31
Unemployed	557	65
Not working due to ill health	32	4
Disclosure status during enrollment (*n* = 948)		
Disclosed	658	69
Not disclosed	290	31
Type of health facility		
Public hospital	563	60
Health center	288	30
Private hospital	98	10

^*^ *Others include Protestant and Catholic religions*

### Clinical characteristics of the study subjects

Only a few (2%) of the patients in the survey had a history of prior exposure to ARVs. Baseline CD4 was determined for 97% of the patients, of whom the majority (69%) had a CD4 count > 100 cells/cm^3^ with a median count of 150 cells/cm^3^ (IQR: 83–222). Nearly half (46%) of the patients started ART based on combined clinical and immunological eligibility criteria. Nucleoside reverse transcriptase inhibitors (NRTIs) containing stavudine and non-nucleoside reverse transcriptase inhibitors (NNRTIs) containing neverapine-based first-line ART were initiated for 528 (56%) and 563 (59%) of the patients, respectively. Restarting first-line ART after interruptions was observed in 140 (15%) of the patients. Poor adherences were recorded in 46 (5%) patients at the time of their least CD4 count record during the course of ART. Almost half (51%) of the patients had a history of regimen change due to toxicity, pregnancy, tuberculosis, etc. Nearly 40% of the patients (*n* = 387) had been on ART for more than 4 years, with a mean ART duration of 44 months (SD: ± 26). Six hundred (60%) of the patients were alive and taking their ART. The majority of the patients (62%, *n* = 594) were at WHO clinical stage III/IV conditions at the time of entry to chronic HIV care, and the percentage was even higher (71%, *n* = 675) at the time of ART initiation (Table 2).

**Table 2:** Clinical characteristics of the study subjects at enrollment to chronic HIV care, initiation of ART, and during follow-up in Dire Dawa, Eastern Ethiopia, January 2014

Clinical characteristics	Frequency	Percent (%)
History of exposure to ARVs before initiation of ART		
Present	19	2
Absent	930	98
Eligibility criteria for ART initiation		
Clinical only	233	25
CD4 only	274	29
Both clinical & CD4	438	46
TLC^*^ & other	4	0
Baseline CD4 (n = 923)		
CD4 > 100 cells/cm^3^	641	69
CD4 ≤ 100 cells/cm^3^	282	31
Median CD4 count = 150 (IQR: 83–222)		
NNRTI-based first-line ART initiated to patients		
Nevirapine based	563	59
Efavirenz based	386	41
NRTI-based first-line ART initiated to patients		
Tenofovir based	285	30
Zidovudine based	136	14
Stavudine based	528	56
Adherence when least CD4 recorded (n = 907)		
Good (> = 95%)	861	95
Poor (< 95%)	46	5
ART interruption and restart history		
Absent	809	85
Present	140	15
History of regimen change (n = 916)		
Absent	469	51
Present	447	49
Duration on ART		
6–24 months	284	30
24–48 months	278	29
≥ 48 months	387	41
Mean duration = 44 months SD: ± 26		
Status of the patient at the time of the survey		
Alive and on ART	600	63
Transfer out	206	22
Lost/drop out	114	12
Dead	29	3
WHO clinical staging at enrollment to pre-ART		
Stage I/II	355	38
Stage III/IV	594	62
WHO clinical staging at ART initiation		
Stage I/II	274	29
Stage III/IV	675	71

^*^ *TLC – total lymphocyte count*

### Magnitude of clinical and immunological failure

**Fig. 1:**
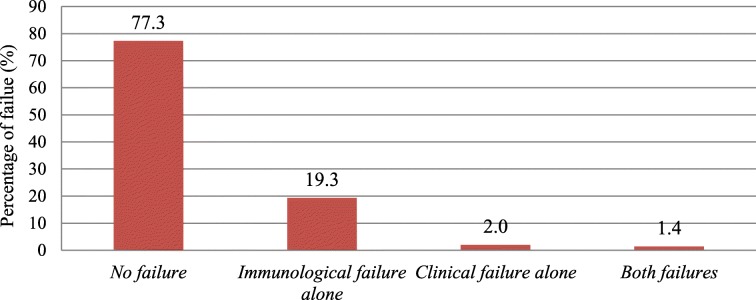
Diagrammatic presentation of the occurrence of clinical and immunological failure among the study subjects in Dire Dawa, Eastern Ethiopia, January 2014

The magnitude of clinical and immunological failure while on first-line ART was 22.7% (95% CI: 20.0–25.4, *n* = 215). Stratifying by the type of failure, immunological failure alone was 19.3% (Fig. 1). Only 33 (15%) of the study subjects with failure status were switched to second-line ART.

**Fig. 2:**
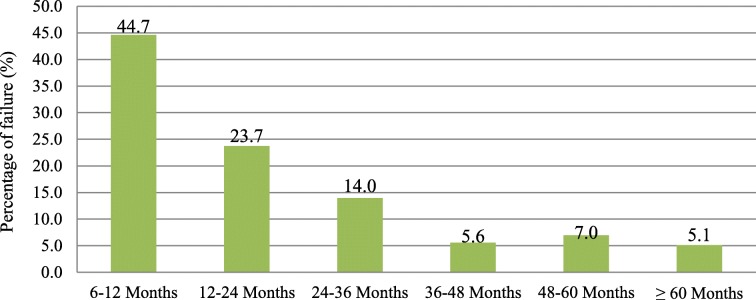
Duration for the occurrence of clinical and immunological failure among study subjects in Dire Dawa, Eastern Ethiopia, January 2014

Ninety-six (45%) of the failures had occurred within 6 to 12 months of ART initiation, and almost 69% of the failures occurred within 6 to 24 months of ART initiation (Fig. 2). The median time for the occurrence of the failure was 14 months (IQR: 8–31).

### Clinical characteristics associated with clinical and immunological failure

The odds of clinical and immunological failure was 1.78 times (AOR = 1.78, 95% CI: 1.18–2.69) higher among study subjects with a baseline CD4 count ≤100 cells/cm^3^ compared to those with CD4 count > 100 cells/cm^3^. Poor adherence was associated with a higher odds of clinical and immunological failure (AOR: 2.5, 95% CI: 1.19–5.25). Similarly, ambulatory or bedridden functional status at the last follow-up visit on ART was associated with higher odds of clinical and immunological failure (AOR: 2.41; 95% CI: 1.12–4.75) (Table 3).

**Table 3:** Multivariate analysis of clinical characteristics associated with clinical and immunological failure in Dire Dawa, Eastern Ethiopia, January 2014

Factors	Failure (%)	No failure (%)	Unadjusted OR (95% CI)	Adjusted OR (95% CI)
Baseline CD4 (n = 923)				
CD4 > 100 cells/cm^3^	125 (19.5)	516 (80.5)	1	1
CD4 ≤ 100 cells/cm^3^	85 (30.2)	197 (69.8)	1.78 (1.29–2.45)	1.78 (1.18–2.69)*
WHO clinical stage when ART initiated (*n* = 949)				
Stage I/II	49 (17.9)	225 (82.1)	1	1
Stage II/IV	166 (24.6)	509 (75.4)	1.50 (1.05–2.13)	0.39 (0.11–1.37)
NRTI-based first-line ART initiated to patients (*n* = 949)				
TDF based	51 (17.9)	234 (82.1)	1	1
ZDV based	30 (22.1)	106 (77.9)	1.29 (0.78–2.18)	1.08 (0.42–2.79)
D4T based	134 (25.4)	394 (74.6)	1.56 (1.09–2.24)	1.19 (0.47–3.05)
Type of health facility (*n* = 949)				
Health center	51(17.7)	237(82.3)	1	1
Public hospital	141(25.0)	422 (75.0)	1.55 (1.09–2.22)	1.21 (0.64–2.28)
Private hospital	23(23.5)	75(76.5)	1.42 (0.82–2.49)	0.94 (0.33–2.65)
Adherence at the least CD4 (*n* = 907)				
Good (> = 95%)	188 (21.8)	673 (78.2)	1	1
Poor (< 95%)	25 (54.4)	21(45.6)	4.26 (2.33–7.78)	2.50 (1.19–5.25)*
Functional status of the patient at last visit on ART (*n* = 949)				
Working	185 (20.8)	703 (79.2)	1	1
Ambulatory/bedridden	30 (49.2)	31 (50.8)	3.68 (2.17–6.23)	2.41 (1.22–4.75)*
History of TB treatment while on ART (*n* = 949)				
Absent	171 (27.7)	655 (72.3)	1	1
Present	44 (35.8)	79 (64.2)	2.13 (1.42–3.20)	1.57 (0.96–2.55)
ART interruption history (*n* = 949)				
Absent	157 (20.1)	652 (79.9)	1	1
Present	58 (41.4)	82 (58.6)	2.94 (2.01–4.29)	1.93 (1.23–3.07)*
History of regimen change (*n* = 916)				
Absent	75 (16.0)	394 (84.0)	1	1
Present	107 (23.9)	340 (76.1)	1.65 (1.19–2.29)	1.50 (1.05–2.15)*
Duration on ART (*n* = 949)				
6–24 months	51 (18.0)	233 (82.0)	1	1
24–48 months	63 (22.7)	215 (77.3)	1.34 (0.89–2.02)	1.44 (0.87–2.38)
≥ 48 months	101 (26.1)	286 (73.9)	1.61 (1.10–2.35)	1.42 (0.79–2.54)
Status of the patient at the time of the survey (*n* = 949)				
Alive on ART	170 (28.3)	430 (71.7)	1	1
Lost/dropped out	34 (29.8)	80 (70.2)	1.54 (0.98–2.40)	1.37 (0.78–2.40)
Transferred out	36 (17.5)	170 (82.5)	0.77 (0.51–1.15)	0.99 (0.63–1.55)
Died	15 (51.7)	14 (48.3)	3.87 (1.82–8.23)	3.94 (1.64–9.45)*

NB: * = *P* < 0.05 and variables that fit in the final model

The presence of restarting treatment after interrupting ART had a 1.93 times (AOR = 1.93, 95% CI: 1.23–3.07) higher odds of clinical and immunological failure compared to those with no similar history. Among study subjects with a history of substitution or change in their first-line ART regimen, the odds of clinical and immunological failure was 1.5 times (AOR = 1.5, 95% CI: 1.05–2.15) higher than those who had no similar history. Compared to study subjects who were alive and on ART, the odds of treatment failure among those who died were almost 4 times (AOR: 3.94, 95% CI: 1.64–9.45) higher (Table 3).

## Discussion

In this study, the magnitude of clinical and immunological failure was 22.7%, with only immunological, only clinical, and both clinical and immunological failures constituting 19.3, 2, and 1.4%, respectively. This magnitude is almost equivalent to the magnitude of failure reported in the Ethiopian study, which is 21% [13]. It is higher than the magnitude reported in the study conducted in Lesotho, South Africa, where the magnitude was 11.8% [17]. It is lower than the magnitude reported in India (26.1%) and Malawi (48%) [18, 19]. Based on the reviewed literature in this study, a very high magnitude of treatment failure is reported from the study in Malawi, but the immunological failure criteria of CD4 drop of 30% from the peak are used. This study used a CD4 count drop of 50% from the peak value as one of the criteria for immunological failure. Other studies also report that with global access to ART increases, it is expected that at least 5–20% of patients will have their first line regimens fail before 4 years of therapy despite adequate adherence, plasma drug levels, and treatment efficacy [20, 21]. This research identifies the median time from the initiation of ART until the occurrence of clinical and immunological failure to be 14 months (IQR: 8–31) and 45% of failures occurring between 6 and 12 months of ART initiation. This finding is less than the finding reported in Ethiopia, where 57.9% of failures occurred during the same period [13]. Another study conducted in Kenya also reported the median time to ART failure to be 37 months (IQR: 15–27) [22]. The Tanzanian cross-sectional study reports 20 months (IQR: 15–27) to be the mean durability of the first-line regimen before failure occurs [23]. The variability in the median time for the occurrence of failure might be explained by the difference in the socioeconomic status between the study areas. The short duration for the occurrence of treatment failure identified in this study goes with the EuroSIDA study that showed a gradual and significant decline in the rate of immunological failure over time [24]. The early occurrence of clinical and immunological failure in this study might be explained by the fact that patients are enrolled in care with severe immune suppression and problem in adherence, as shown in the factor analysis. As indicated in Table 2, the median baseline CD4 count in this study is 150 cells/mm^3^ compared to the baseline CD4 count of 200 cells/mm^3^ in the EuroSIDA study [24].

Among patients identified as failure, only 15% were switched to second-line ART. This is far less than the record in the Tanzania study, where 41% of failed patients initiated second-line ART [23]. This might show that patients who are failing on the first-line regimen are not switched in a timely manner to the second-line regimen. Studies have shown that putting patients on a failing first-line regimen leads to high morbidity and mortality [10, 25, 26]. An indication of this fact in this study is that among study subjects who died and had clinical and immunological failure, only 6.7% of them were switched to second-line ART, and patients who died were 4 times (AOR: 3.9495, 95% CI: 1.64–9.45) more likely to have clinical and immunological failure compared to those who were alive and on treatment at the time of the study. This might be due to the reduced attention given to diagnosing failure early, lack of trained manpower to pick and manage treatment failures early, and lack of early and accurate diagnostic setup. Early initiation of a second-line regimen after the diagnosis of failure is a key for the reduction of morbidity and mortality associated with taking a failing regimen [15].

This research identifies that patients with a baseline CD4 count of ≤100 cells/mm^3^ have 1.78 (95% CI: 1.18–2.69) times higher odds of clinical and immunological failure compared to patients with a CD4 count greater than 100 cells/mm3. Similar findings are observed in the studies conducted in Ethiopia and Tanzania [13, 23]. The report in the Kenyan study showed a CD4 count of < 50 cells/mm3 to be a significant predictor for treatment failure [22]. This finding might be because patients with a baseline CD4 count of ≤100 cells/mm3 have reduced immunity, and the response to the first-line ART may be unsatisfactory.

This study shows that poor adherence is independently associated with clinical and immunological failure (AOR: 2.5; 95% CI: 1.19–5.25). This is similar to findings from the report in the Kenya study, which showed imperfect adherence to be an independently associated factor for treatment failure (AOR: 2.77; 95% CI: 2.20–3.49) [22]. Other studies also show similar findings [23, 27, 28]. Adherence to ART is needed to ensure optimal benefit from the ARVs [29]. Treatment interruption and restarting the first-line ART again is also a form of non-adherence, and this research study found that patients with a history of restarting treatment after interruption have a 1.5 times (AOR: 1.93, 95% CI: 1.23–3.07) higher odds of clinical and immunological failure than those who never had a similar history. Even if the criteria for failure are different from this study, treatment interruption is reported to be a significant predictor of virological failure in the Gabon study [30]. A study in Uganda also reported that ARV resistance was reported in patients who interrupted their therapy [31].

The functional status of the patient at their last follow-up visits was assessed, and the finding showed that patients who were “ambulatory or bedridden” had a 2.4 times (AOR: 2.41, 95% CI: 1.22–4.75) higher odds of clinical and immunological failure. This might be due to the higher rate of morbidity associated with the inability to identify failures early and switch to a second-line regimen, resulting in the occurrence of different opportunistic infections and other diseases, which in turn leads to impaired functional status. Baseline functional status shows no significant associations, which is similar to the report in the Ethiopian study [13].

Regimen change/substitution of the original first-line ART to an alternative first-line ART for different reasons is associated with a 1.5 times (AOR: 1.50, 95% CI: 1.05–2.15) higher odds of clinical and immunological failure compared to those with no history of regimen change. The EuroSIDA study reports that a substitution to the ART regimen showed marginal statistical significance (AHR: 1.21, 95% CI: 0.98–1.48) [24]. This finding might show the presence of substitution of first-line ART regimens, which may lead to the occurrence of resistance and an increasing chance of treatment failure.

### Limitation of the study

The study uses secondary data collected retrospectively resulting in incompleteness of clinical data, and hence, information bias may have occurred because of underreporting/missing data elements. The magnitude of clinical and immunological failure could be overestimated as no confirmatory virological tests were performed to confirm the failure because the use of immunological and clinical failure criteria lacks both sensitivity and specificity to detect failure.

## Conclusion

There was a high magnitude of clinical and immunological failure. Nearly half of the failures occurred during the first 12 months of ART initiation. Switching to second-line ART was low. A very low baseline CD4 count was associated with a high occurrence of failure. Higher odds of failures were also observed in patients with poor adherence. Interrupting and restarting of the first-line ART were associated with higher odds of failure. Substituting/changing a first-line regimen with an alternative first-line regimen was also a factor associated with a high occurrence of clinical and immunological failure. The presence of ambulatory or bedridden functional status at the last follow-up visit on ART might show that there was a significant level of morbidity due to clinical and immunological failure. Failure was also present significantly among patients who died, showing the high magnitude of mortality associated with the use of probably a failing first-line ART.

Early diagnosis of HIV-positive status, early enrollment in chronic HIV care, and initiation of ART before the occurrence of severe immune suppression should be promoted. Early recognition and management of clinical and immunological failure should be given due attention. Efforts to improve adherence to therapy should be strengthened. Regimen change or substitution to the original first-line ART regimen should be made cautiously. Despite some limitations, this study is a major step forward for the regional and national HIV program and should serve as an impetus for further studies using primary data in this area.

## Data Availability

The datasets (SPSS) used and analyzed during the current study are available from the corresponding author on reasonable request.

## Abbreviations

AHR: Adjusted Hazard Ratio
AIDS: Acquired Immunodeficiency Syndrome
AOR: Adjusted Odds Ratio
ART: Antiretroviral Therapy
ARVs: Antiretroviral drugs
CI: Confidence Interval
FHAPCO: Federal HIV/AIDS Prevention and Control Office
FMoH: Federal Ministry of Health
HIV: Human Immunodeficiency Virus
ICT: Information Communication Technology
IQR: Inter Quartile Range
NNRTI: Non-Nucleoside Reverse Transcriptase Inhibitor
NRTI: Nucleoside Reverse Transcriptase Inhibitor
OIs: Opportunistic Infections
PI: Protease Inhibitor
PLHIV: People Living with HIV
SPSS: Statistical Package for the Social Sciences
SSA: Sub-Sahara Africa
TB: Tuberculosis
UNAIDS: The United Nations Joint Program on HIV/AIDS
UNICEF: United Nations International Children’s Emergency Fund
WHO: World Health Organization
